# The relationship between empowering leadership and radical creativity

**DOI:** 10.3389/fpsyg.2022.1002356

**Published:** 2022-10-10

**Authors:** Wenjun Yin, Su Liu

**Affiliations:** ^1^School of Economics and Management, Hubei University of Science and Technology, Xianning, China; ^2^School of Management, Huazhong University of Science and Technology, Wuhan, China

**Keywords:** empowering leadership, radical creativity, job control, willingness to take risks, error management climate

## Abstract

Drawing on the conservation of resource theory, we theorized and tested a serial mediation model linking empowering leadership with employee radical creativity through job control and willingness to take risks. We tested our hypotheses using data collected from a time-lagged and multisource survey of 385 employees in 84 research and development teams from 20 different companies. The results demonstrated that empowering leadership had a positive indirect effect on employee radical creativity *via* job control and willingness to take risks, and the error management climate was found to strengthen this indirect effect. Theoretical and practical implications are also provided in the discussion section.

## Introduction

Radical creativity, referring to “ideas that differ substantially from an organization’s existing practices” (Madjar et al., 2011), is an innovation engine and a critical source of development and competitiveness that can bring considerable benefits to organizations (Shalley et al., 2004). Due to the significance of radical creativity for companies in the current dynamic and competitive environment, researchers and practitioners have begun to explore the antecedents and practices that can predict employees’ radical creativity (Gong et al., 2017; Tang et al., 2017). Previous studies have found personal factors, such as intrinsic motivation (Malik et al., 2019), experiencing tensions (Liu et al., 2022), and openness to experiences (Xu et al., 2018), and contextual factors, such as structural holes of knowledge networks (Tang et al., 2017) and social network ties of employees’ immediate leaders (Venkataramani et al., 2014), have positive effects on employee radical creativity.

However, research into the effect of leadership on employee radical creativity has been very limited. While, leadership has long been considered an essential antecedent of individual creativity (Qu et al., 2015; Hughes et al., 2018), only a few empirical studies have focused on the impact of specific types of leadership, such as paternalistic leadership (Wang et al., 2019) and supportive supervision (Gilson et al., 2012), on employee radical creativity. Notably missing from research attention has been empowering leadership, despite it has been proved having a positive impact on creativity (e.g., Zhang and Bartol, 2010; Harris et al., 2014; Zhang and Zhou, 2014; Zhang et al., 2018). Empowering leadership aims to promote employees’ psychological empowerment and self-leadership through three core processes: power sharing, motivational support and developmental support (Amundsen and Martinsen, 2014). Earlier studies have proved that empowering leadership can influence employee creativity by improving employees’ intrinsic motivation (Zhang and Bartol, 2010) and creative self-efficacy (Zhang and Zhou, 2014). However, previous researches on the relationship of empowering leadership on employee creativity failed to address two important issues.

First, prior researches have all treated creativity as a very general and homogeneous construct, ignoring scholars’ calls to address specific forms of creativity (Unsworth, 2001). Because scholars have proved that creativity can range from minor changes to radical breakthroughs (Mumford and Gustafson, 1988; Shalley et al., 2004), and that makes the antecedents and mechanisms leading to different forms of creativity vary (Malik et al., 2019). As mentioned above, radical creativity tends to be disruptive (Madjar et al., 2011), which is distinctive with other forms of creativities. Therefore, the mechanism linking empowering leadership with employee radical creativity can not be confused with the mechanism linking empowering leadership with employee general creativity, which has been ignored in the existing studies.

To fill this gap, drawing on the conservation of resource (COR) theory (Hobfoll, 1989), we propose that empowering leadership can influence employee radical creativity *via* job control and willingness to take risks. As known, instigating employee radical creativity requires considerable psychological resources (Zhang and Bartol, 2010; Chen et al., 2011; Zhang and Zhou, 2014), while empowering leadership, offering authority and external resources to employees, can enhance their internal resources (i.e., job control, referring to “the belief that one can exert some influence over the environment, either directly or indirectly, so that the environment becomes more rewarding or less threatening,” Aryee et al., 2013, 797). After being empowered and experiencing a high degree of job control, employees would obtain psychological resources to defend against the risk of failure, which may increase their willingness to take risks, which refers to one’s willingness to take potential risks at work, striving to achieve positive organizational outcomes while also having an open attitude to the possibility of adverse personal outcomes (Dewett, 2006). Moreover, willingness to take risks is crucial for engaging in radical creativity because radical creativity, representing a challenge and disruption to the current status quo, is risky (George, 2007). Hence, we propose job control and willingness to take risks are the precise dynamics linking empowering leadership with employee radical creativity.

Second, majority of existing researches often invest the boundary effect of employee’s personal trait on the relationship of empowering leadership on employee creativity, such as empowerment role identity (Zhang and Bartol, 2010) and uncertainty avoidance (Zhang and Zhou, 2014), few has discussed the boundary effect of organizational factor (i.e., perceived organizational support for creativity, Harris et al., 2014), but have focused on the individual level. Prior researches have rarely considered how the group climate influence the effect of empowering leadership on employee creativity, especially on the group level. However, plenty of existing researches have proved that group climate could superimpose or substitute the effect of leadership on employee creativity (Hughes et al., 2018), that is valuable to explore.

With regard to forementioned, filling this gap, we focus on the boundary effect of error management climate (EMC), referring to employees’ common perceptions of their group’s practices related to dealing with errors and their consequences (Van Dyck et al., 2005; Cigularov et al., 2010). Preceding has expounded that radical creativity involves high levels of uncertainty and low levels of predictability (Madjar et al., 2011), which means errors are bound to creep in during the process. Thus, the attitudes toward errors in the work units (organization and/or group) may influence employees’ perceptions of the work unit, which in turn may affect whether employees choose to invest in radical creativity. We propose that EMC could moderate the linkage between job control and willingness to take risks, thereby serving to moderate the indirect effects of empowering leadership on radical creativity.

The present study contributes to the existing knowledge base in several ways. First, this research extends the antecedents of work carried out to investigate radical creativity by exploring the effect of empowering leadership behavior. Previous research has focused on factors that predict employee radical creativity, but less is known about the effect of leadership. This study aims to supplement the existing knowledge with insights about the influence of leadership on employee radical creativity. Second, based on the COR theory, the research examines and explains a dynamic process linking empowering leadership and employee radical creativity, describing how the resources invested by leaders increase employees’ personal resources and strengthen their courage to explore less routine perspectives. Finally, this research contributes to the empowering leadership literature by exploring how the group climate superimpose the effect of empowering leadership on employee’s creative behavior. Specifically, we argue that a team EMC might enhance employees’ psychological process of transforming and accumulating personal resources, obtained from empowering leadership, and then motivate them to engage in radical creativity. This research provides important guidance on how to utilize team contextual factors to amplify the effectiveness of empowering leadership on employee radical creativity.

## Theory and hypotheses

### Empowering leadership and job control, willingness to take risks

Empowering leadership delegates and shares working power to employees, by strengthening the significance of work to employees, encouraging and cultivating employees’ participation in work decisions, expressing affirmation of employees work performance, and offering job autonomy power to employees (Arnold et al., 2000; Ahearne et al., 2005; Zhang and Bartol, 2010). Previous studies have revealed that empowering leadership can have a positive impact on employees’ attitudes and behaviors through enhancing the psychological resources of employees in the workplace. For instance, scholars found that psychological empowerment (Zhang and Bartol, 2010; Chen et al., 2011, 2019; Lorinkova and Perry, 2017), self-efficacy, (Arnold et al., 2000; Ahearne et al., 2005; Cheong et al., 2016) and intrinsic motivation (Zhang and Bartol, 2010) are important explaining mechanisms of empowering leadership on employees’ work outcomes, such as job satisfaction (Arnold et al., 2000; Vecchio et al., 2010), in-role performance (Ahearne et al., 2005; Chen et al., 2007), organizational citizenship behavior (Li et al., 2017) and creativity (Zhang and Bartol, 2010; Chen et al., 2011; Harris et al., 2014). Therefore, we infer that empowering leadership would influence employees’ behaviors through enriching their psychological resources.

Job control refers to a perceived ability to influence the work environment making it more rewarding and less threatening (Bond and Bunce, 2003; Aryee et al., 2013). Some scholars argue that job control reflects the degree of an employee’s discretion at work, including their possibilities to use technology and knowledge, their opportunities to participate in decision-making (Karasek, 1990; Elovainio et al., 2001), and their ability to influence their work environment (Demerouti et al., 2001a). Previous studies have shown that offering job autonomy and flexibility to employees may increase their job control. For example, researchers found that family supportive supervisor would increase an employee’s job control by delegating to them the responsibility to flexibly arrange their own working time and locations (Thomas and Ganster, 1995; Aryee et al., 2013). The formulation and implementation of these work practice policies are usually determined by leaders, which means their thoughts and behaviors would probably impact employee job control. Therefore, we propose empowering leadership, with an emphasis on delegation to, and enabling, employees, would have positive effect on employees’ job control.

According to the COR theory (Hobfoll, 1989), empowering leadership that provided job autonomy to employees would be interpreted as offering them working resources, which may transform into their own resources and enhance their psychological resources. To be specific, first, empowering leadership can improve employees’ perception of the identification and valuing of their work, by strengthening their sense of the meaning of their work, which may increase employees’ perceived psychological rewards from their work (Kahn, 1990). This perception of the significance of their work would establish and enhance their competence at work and strengthen their sense of job control. Second, empowering leadership provide their skills and perspectives as resources for employees to learn and apply, through encouraging and cultivating employees to participate in work decision-making. This will enrich and broaden employees’ ability to control their work, including improving their work content and methods. Third, empowering leadership can enhance employees’ positive psychological resources (Cheong et al., 2016), such as confidence, optimism, and self-efficacy, through expressing affirmation and their belief in their employees’ high performance, enabling employees to feel confident in their work abilities. However, self-efficacy is a key element to improve an individual’s sense of control (Litt, 1988). Finally, empowering leadership delegate and share discretionary power resources to employees, enabling them to arrange their work schedule and allocate work resources according to their needs, thus improving their job control. All in all, we predict that through delegating and enabling, empowering leadership have a positive effect on employee job control. Thus, we propose:

*Hypothesis 1 (H1)*: Empowering leadership is positive related to employee job control.

Willingness to take risks refers to the willingness of employees to undertake potential risks at work in striving to achieve positive organizational results, while keeping an open mind about the possible negative impacts on themselves (Dewett, 2006). We infer that a sense of control may increase the psychological resources necessary for employees to undertake risks, in turn increasing their willingness to take risks. Job control is a positive psychological resource, helping individuals to resist pressure (Hobfoll, 1989, 2002). Previous research has also shown that job control can effectively alleviate negative emotions from risk at work (Greenberger and Strasser, 1986; Ganster and Fusilier, 1989; Schaubroeck and Merritt, 1997). Therefore, employees with a high degree of job control would possess more resources to protect themselves, helping them resist harm and failure more successfully. This may decrease their hesitation when considering risky behaviors, increasing their willingness to take risks. Additionally, researchers have shown that employee job control is highly related with work involvement and affective commitment (Demerouti et al., 2001b; Salanova et al., 2005). If employees identify strongly with and committed to their organization, they would likely be more motivated to take risks to benefit their organizations. For example, employees will proactively report their errors, if they have more consideration for their organization (Zhao and Olivera, 2006). According to the definition, a willingness to take risks is a motivation for contributing to organizational benefit. Therefore, we infer that employees’ job control may enhance their affective attachment to an organization, and increase their willingness to take risks for organizational benefit. Thus, we propose:

*Hypothesis 2A(H2A)*: Employee job control is positive related to willingness to take risks.

As aforementioned, after receiving power from empowering leadership, employees may perceive confidence and efficacy in controlling their work, increasing their own psychological resources. This can enhance their resilience upon suffering defeat and then increase their motivation to engage in risky behavior for organizational benefit. Thus, we assume job control to be a psychological mechanism explaining how empowering leadership can positively influence employees’ willingness to take risks. Therefore, we propose:

*Hypothesis 2b (H2b)*: Employee job control mediates the positive relationship between empowering leadership and employee willingness to take risks.

### Willingness to take risks and radical creativity

Radical creativity emphasizes novelty (Litchfield, 2008; Litchfield et al., 2015), disrupting established processes and frameworks (Gilson and Madjar, 2011), which means breaks the existing balance (Albrecht and Hall, 1991). Hence, radical creativity is always viewed as being highly uncertain and risky (George, 2007). We infer that employees with a high level of willingness to take risks are more likely to engage in radical creativity. Willingness to take risks is essential for employees to become persistently involved in any risky working behaviors for their organizations. Researchers have found that willingness to take risks is one of the core cultural characteristics in some successful technology companies (Abbey and Dickson, 1983). Employees need to be strongly motivated to face and undertake risks so that they can continue to engage in radical creativity, due to the unconventional nature of radical creativity, which makes employees cannot clearly predict or control the processes or their results and may be more likely to fail. Therefore, employees with a high level of willingness to take risks are more likely to invest in and improve their radical creativity. Additionally, employees with a willingness to take risks are rational, rather than blindly, emotionally impulsive, for having assessed the risks and pressures they would suffer, they would make the same choice (Dewett, 2006). Thus, employees with a high level of willingness to take risks are more likely to capture subtle and unconventional ideas, from the surrounding environment and from a wide range of information sources, less concerned with errors and failure that could constrain the breadth and width of their thought. Therefore, they have more potential to generate radical creativity.

Thus, we propose:

*Hypothesis 3(H3)*: Employee willingness to take risks is positive related to radical creativity.

Taken as a whole, the prior hypotheses imply an indirect effect model. Specifically, we proposed that empowering leadership would be associated with willingness to take risks *via* job control, and that willingness to take risks is related to radical creativity. Based on our earlier discussion, willingness to take risks is thus proposed to be a mechanism by which empowering leadership and job control relate to radical creativity. Thus, we propose:

*Hypothesis 4(H4)*: Empowering leadership has a positive indirect relationship with employee radical creativity, via job control and willingness to take risks.

### The moderating role of the EMC

The EMC, referring to employees’ common perceptions of a team’s practices in relation to error communication, error competence, learning from errors, and thinking about errors (Van Dyck et al., 2005; Cigularov et al., 2010), reflects a team’s attitudes when dealing with errors. It focuses on reducing the negative impact of errors and increasing their positive effects (Van Dyck et al., 2005). Previous research suggests EMC can have a positive effect on employees’ extra-role behavior and performance, such as error reporting (Gronewold et al., 2013), employee voice (Cheng et al., 2022), and innovation performance (Edmondson, 2004), as it enhances employees’ consideration of safety and reduces the costs and burdens of engaging in behaviors that are risky but beneficial to the organization. Accordingly, we infer that the EMC can moderate the effect of employees’ job control on their willingness to take risks.

Researchers have shown that EMC can effectively weaken the negative consequences of errors (Cigularov et al., 2010). At a high level of EMC, the team tolerates errors, and employees perceive that their team can understand and accept the errors that inevitably accompany risky behaviors but that this will benefit the team to a certain extent (Van Dyck et al., 2005). In this context, employees may suffer less pressure and require fewer resources to resist the pressure. Based on the COR theory, individuals will have greater motivation to access new resources, when they need fewer resources to withstand pressure (Hobfoll, 2002). Hence, at a high level of EMC, employees will have less concern about the pressure of taking risks for the team, and that would amplify the effect of their sense of job control on their willingness to take risks. At a low level of EMC, however, the team focuses on preventing errors from occurring, severely punishing employees when they make an error. Employees will have a psychological burden and utilize more resources when engaging in risky behavior, which would increase the difficulties and costs they anticipated before taking the action, suppressing and weakening their willingness to take risks. Therefore, in this situation, the effect of employees’ job control on their willingness to take risks will be weakened. Thus, we propose:

*Hypothesis 5A (H5A)*: The EMC strengthens the relationship between job control and willingness to take risks.

As discussed above, employee radical creativity peaks when employee has perceived high level of empowering leadership. After being empowered, employees whose group has a high level of EMC, would have more energy and courage to take adventure, consequently generating unconventional ideas. Additionally, a group with high level of EMC, is always regarded as innovation oriented (Frese and Keith, 2015; Chen et al., 2021; Cheng et al., 2022). Therefore, integrating our inference in the current section with our earlier theorizing, we consider that the indirect effect of empowering leadership on employee radical creativity can be superimposed on the group climate of tolerating errors. To sum up, we predict that the EMC will moderates the indirect of empowering on employee radical creativity through job control and willingness to take risks. Thus, we propose:

*Hypothesis 5B (H5B)*: The EMC strengthens the indirect effects of empowering leadership on employee radical creativity, via job control and willingness to take risks.

**Figure 1:**
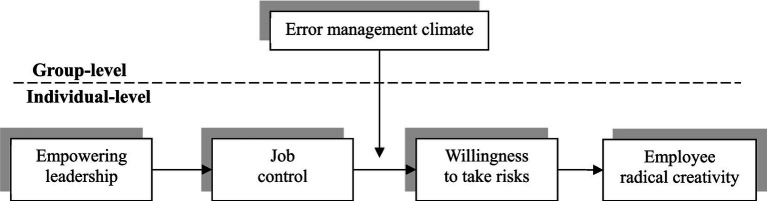
Proposed theoretical model.

Based on the COR theory, we predict an indirect effect from empowering leadership to employee radical creativity through job control and willingness to take risks and examine how EMC moderates this mechanism. A conceptual model is shown in Figure 1.

## Materials and methods

### Sample and procedure

To test our hypotheses, we randomly recruited 20 students from two EMBA classes of a Double First-Class university in Wuhan to participate in the survey. These students are all senior managers or executive directors of their enterprises, so they can provide sufficient opportunities and resources for researchers to collect data. Their companies are from cities in mainland China, including Beijing, Changsha, Shenzhen and Wuhan. The industries include information technology (IT), metallurgy, automobile manufacturing, biomedical companies and real estate. In each of these 20 companies, we randomly recruited five R&D teams to participate in the survey. The primary reason we chose R&D teams is that there is a more obvious correlation between the work content of technical positions and innovation, so the participants are more sensitive to creativity compared with other job areas, and this would be more easily reflected in the survey results. With the assistance of the directors of each R&D department and human resources managers, we assigned each team member and their leaders a numerical code (the coding rule was: leader code = “team member”; member code = “team member-participant number”).

We collected the data in 2020; to reduce common-method bias, we conducted three waves of data collection, with a 2-month time lag between each successive time point. At Time 1, the team members were asked to report their demographic characteristics, including gender, age, education level and organizational tenure, as control variables, as well as their evaluation of empowering leadership. Two months later, at Time 2, all team members were asked to evaluate their sense of job control and willingness to take risks, as well as their perception of the EMC in their team. After 8 weeks, at Time 3, team leaders were asked to complete a questionnaire assessing the radical creativity of their members.

A total of 480 team members from 100 teams were asked to complete the questionnaires at the beginning of the survey. We obtained valid responses from 385 team members (80.2% valid response rate) from 84 teams (84.0% valid response rate), after removing those invalid questionnaires lacking completing three-phase responses or with the same score for all items. From the final sample, the mean number of members per team was 4.58. Of the 385 team members, 51.7% were male, with an average age of 30.65 years (SD = 5.52) and an average of organizational tenure of 4.51 years (SD = 4.73). They were highly educated: 58.2% had a bachelor’s degree and 19.0% had obtained a master’s degree or higher.

### Measures

All the scales we used were initially written in English. By using a common back-translation process (Brislin, 1986), we translated all the items into Chinese. Except for control variables, all the variables were measured on seven-point Likert scales ranging from 1 (strongly disagree) to 7 (strongly agree).

#### Empowering leadership

A 12-item scale developed by Ahearne et al. (2005) was used in the present study. Each team member was asked to evaluate the extent to which they perceived their team leaders’ empowering behaviors. A sample item was “My leader makes many decisions together with me.” (Cronbach’s *α* = 0.92).

#### Perceived control

An 11-item scale developed by Van Yperen and Hagedoorn (2003) was used in the present study. Each team member was asked to evaluate their sense of job conditions. A sample items was “I can plan my own work” (Cronbach’s *α* = 0.89).

#### Willingness to take risks

A two-item scale developed by Schilpzand et al. (2018) was used in the present study. Each team member was asked to assess the extent to which they were willing to take a risk with their work. A sample item was “I will take a risk and try something new that might improve work” (Cronbach’s *α* = 0.79).

#### Radical creativity

A three-item scale developed by Madjar et al. (2011) was used in the present study. Each team leader was asked to assess their team members’ performance in terms of radical creativity. A sample item of radical creativity was “This team member demonstrates originality in his/her work” (Cronbach’s *α* = 0.91).

#### EMC

A 16-item scale developed by Cigularov et al. (2010) was used in the present study. Each team member was asked to assess their perception of how their team treated and managed errors. A sample item of the EMC was “Our errors point us at what we can improve” (Cronbach’s *α* = 0.94). We computed *r_wg_\*, ICC(1) and ICC(2) to assess whether this variable was appropriate for aggregation to the team level. The results showed an acceptable within-team agreement and a qualified intraclass correlation coefficient (average *r_wg_\* = 0.98, ICC[1] = 0.36, ICC[2] = 0.73), supporting the aggregation of the EMC.

#### Control variables

We controlled the demographic variables such as employee gender, age, educational level and organizational tenure, as these variables have been found to exert an influence on employee radical creativity (Gong et al., 2017).

## Results

### Confirmatory factor analyses, descriptive statistics, and correlations

We conducted a series of confirmatory factor analyses (CFA) using Mplus 8.3 to examine the discriminant validity of our measures. Results indicated that our measurement model with five factors, including empowering leadership, job control, willingness to take risks, radical creativity, and the EMC, showed a significantly better fit (*χ*^2^ [109] = 378.91; CFI = 0.94, TLI = 0.92; RMSEA = 0.08) than a four-factor model grouping independent variables and mediating variables (i.e., empowering leadership and job control: *χ*^2^ [113] = 828.90; CFI = 0.83, TLI = 0.80; RMSEA = 0.13), and another four-factor model grouping two mediating variables (i.e., job control and willingness to take risks: *χ*^2^ [113] = 700.44; CFI = 0.86, TLI = 0.83; RMSEA = 0.12), suggesting the distinctiveness of these variables.

Table 1 shows the means, standard deviations, and correlation coefficients among the variables in this study.

**Table 1:** Means, standard deviations, correlations, and reliabilities.

Individual-level variables	Mean	SD	1	2	3	4	5	6	7	8	9
1. Gender	0.48	0.50	–								
2. Age	30.65	5.52	0.07	–							
3. Education	3.96	0.69	−0.13^*^	−0.19^**^	–						
4. Tenure	4.52	4.73	0.01	0.65^**^	−0.16^**^	–					
5. Empowering leadership	5.27	0.83	0.05	0.01	−0.05	−0.01	(0.92)				
6. Job control	5.04	0.83	0.00	0.02	0.03	0.01	0.49^**^	(0.89)			
7. Willingness to take risks	4.76	0.85	0.11^*^	−0.10^*^	−0.11^*^	−0.06	0.31^**^	0.38^**^	(0.79)		
8. Radical creativity	4.64	1.08	0.16^**^	−0.06	0.08	−0.07	0.40^**^	0.50^**^	0.46^**^	(0.91)	
**Group-level variables**											
1. EMC	5.36	0.54	–								(0.94)

*N* = 385, *n* = 84; Cronbach’s alphas were reported along the diagonal. ^*^p < 0.05; ^**^p < 0.01.

### Hypothesis testing

To test the research hypotheses, we conducted a multilevel path analysis in Mplus 8.3, in which empowering leadership was included as the independent variable, job control and willingness to take risks were included as the mediators, radical creativity was included as the dependent variable, and EMC was included as the moderator. The results are displayed in Table 2. Hypothesis 1 proposed the direct effect of empowering leadership on employee job control. As shown in Table 2, the result revealed that empowering leadership was positive related to employee job control (*b* = 0.46, *p* < 0.01), supporting Hypothesis 1. In keeping with Hypothesis 2A, employee job control was found to significantly influence willingness to take risks (*b* = 0.23, *p* < 0.01). Hypothesis 2B suggested that employee job control mediated the effect of empowering leadership on employee willingness to take risks. The bootstrapping approach with 20,000 replications revealed that the indirect effect of empowering leadership on employee willingness to take risks *via* job control was significantly positive (*indirect effect* = 0.11, 95% *CI* = [0.05, 0.17]), thus supporting Hypothesis 2B.

**Table 2:** Results of the path analysis.

Independent variables	Mediators			Dependent variables		
	Job control		Willingness to take risks		Radical creativity	
Individual-level	Estimate	S.E.	Estimate	S.E.	Estimate	S.E.
Gender	−0.07	0.08	0.07	0.08	0.17	0.10
Age	−0.00	0.01	−0.02	0.01	−0.01	0.01
Education	−0.02	0.08	−0.15	0.08	0.13	0.09
Tenure	0.01	0.01	0.01	0.01	−0.01	0.02
Empowering leadership	0.46^**^	0.05	0.22^**^	0.07	0.15	0.09
Job control			0.23^**^	0.06	0.42^**^	0.09
Willingness to take risks					0.42^**^	0.08
**Group-level**						
EMC			0.01	0.20		
**Cross-level interaction**						
Job control × EMC			0.08^*^	0.04		

*N* = 385, *n* = 84; ^*^p < 0.05; ^**^p < 0.01; Unstandardized coefficients are reported.

Hypothesis 3 proposed the direct effect of employee willingness to take risks on radical creativity. The result, as displayed in Table 2, revealed that employee willingness to take risks has significantly positive effect on radical creativity (*b* = 0.42, *p* < 0.01), supporting Hypothesis 3. Hypothesis 4 stated that there was a positive indirect relationship between empowering leadership and employee radical creativity *via* job control and willingness to take risks. Following aforementioned procedures, bootstrapping result indicated that the indirect relationship between empowering leadership and employee radical creativity *via* job control and willingness to take risks was significant and positive (*indirect effect* = 0.04, 95% *CI* = [0.02, 0.08]), supporting Hypothesis 4.

**Figure 2:**
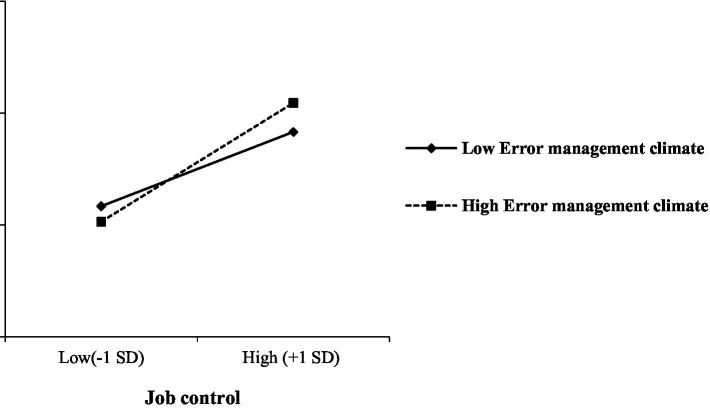
Interaction between Job control and EMC on willingness to take risks.

Hypothesis 5A predicted that EMC moderates the effect of job control on willingness to take risks, such that the positive effect of job control on willingness to take risks become stronger as EMC higher. Table 2 showed that the cross-level interaction term for EMC and job control was significant and positive (*b* = 0.08, *p* < 0.05). Figure 2 displayed the plot of this interaction. We further examined the simple slope test. The result indicated that when EMC is high, job control was positively related to willingness to take risks (*simple slope* = 0.27, *p* < 0.01) and when EMC is low, job control was positively related to willingness to take risks (*simple slope* = 0.18, *p* < 0.01). Additionally, the difference between the two conditional indirect effects was significant (*simple slope* = 0.08, *p* < 0.05), thus supporting Hypothesis 5A.

Meanwhile, Hypothesis 5B proposed that EMC strengthens the indirect effects of empowering leadership on employee radical creativity, *via* job control and willingness to take risk. The bootstrapping results revealed that the conditional indirect effect of empowering leadership on employee radical creativity was significant when EMC was low (*indirect effect* = 0.04, 95% *CI* = [0.01, 0.07]), and also significant when EMC was high (*indirect effect* = 0.05, 95% *CI* = [0.02, 0.09]). Additionally, the difference between the two conditional indirect effects was significant (*difference* = 0.02, 95% *CI* = [0.00, 0.04]). Thus, Hypothesis 5B received support (Appendix).

## Discussion

The present study explored and examined a theoretical model to explain how empowering leadership can positively affect employee radical creativity. Consistent with our prediction, all the hypotheses are supported. The results revealed that empowering leadership exerted a positive indirect effect on employee radical creativity *via* job control and willingness to take risks. Additionally, the EMC moderated the relationship between job control and willingness to take risks, such that the positive effect was stronger when the EMC increased. We also found that the beneficial effect of empowering leadership on employee radical creativity *via* job control and willingness to take risks could be attenuated when the EMC was high. Our findings offer several theoretical and practical implications.

### Theoretical implications

Our findings make some useful theoretical contributions to the existing research.

First, increasing numbers of researchers have called for an end to the use of a general concept of creativity in research, a widely recognized dualistic variable (*cf.* radical creativity and incremental creativity; Unsworth, 2001; Gilson and Madjar, 2011; Madjar et al., 2011). Responding to this call, the present study focused on the antecedents of radical creativity, examining the positive relationship between empowering leadership and employee radical creativity, which to fill the knowledge gap relating to the effects of leadership on employee radical creativity. Specifically, the results showed that empowering leadership can improve employee radical creativity, verifying previous views on the effect of empowering leadership on employee creativity (Zhang and Bartol, 2010), and enriched the research perspectives on the antecedents of radical creativity.

Second, this study revealed the mediating role of employees’ willingness to take risks, offering a new perspective on research into the effect of empowering leadership on employee radical creativity. Previous studies on the mediating mechanism of empowering leadership and employee creativity have mainly focused on psychological empowerment (Zhang and Bartol, 2010; Li et al., 2017; Lorinkova and Perry, 2017), self-efficacy (Arnold et al., 2000; Cheong et al., 2016) and intrinsic motivation (Zhang and Bartol, 2010), neglecting the possible linking mechanism from the perspective of risk taking. This may be due to the use of the general concept of creativity, masking the concept of incremental creativity, not being seen as a high-risk behavior. Due to its high degree of uncertainty and subversion of conventional frameworks, radical creativity is a considerably high-risk behavior (Gilson and Madjar, 2011), which requires employees to be prepared to take risks. Therefore, this study explored and verified that employees’ willingness to take risks serves as an explanatory mechanism for empowering leadership and employee radical creativity, extending previous research.

Finally, this study enriched the knowledge base from studies on the boundary conditions of the impact of empowering leadership on employee creativity. Reviewing previous studies in this field, mainstream researches have focused on employee traits (Zhang and Bartol, 2010) and the interaction relationships between employees and leaders (Harris et al., 2014), as the boundary conditions, ignoring the influence of group climate. Our findings introduced the EMC as a group situational factor, and explored and verified its moderating effect on the effect of empowering leadership on employee radical creativity, enriching the existing research results that the effectiveness of empowering leadership can be superposed by suitable group climate.

### Practical implications

Our findings also have some practical implications. First, from the perspective of power distribution, a flat team structure and an authorized management style, rather than a centralized team structure and a direct imperative management style, may be more conducive to promoting employee radical creativity. Leaders can enhance employees’ sense of control over their job and work environment by delegating some responsibilities and powers to employees, enhancing their job discretion, reducing the hierarchy in the team, and expanding their scope of authority, which in turn further stimulates employee radical creativity.

Second, from the perspective of team member management, enhancing employees’ sense of ownership may be more conducive to enhancing their radical creativity. When employees have high affective commitment to their organization, they are more likely to engage in risky but beneficial behaviors for that organization. Therefore, leaders can improve employee radical creativity by encouraging employees to participate more in decision-making and by treating employees’ work with a positive attitude, such as encouragement and praise. This promotes employees’ affective commitment to the team and strengthens their willingness to strive for the benefit of the organization, regardless of personal gain or loss.

Finally, from the perspective of fostering team culture, a positive error management climate in a team is more conducive to increasing the efficiency of empowering leadership and fostering employee radical creativity. A relaxed environment is more suitable for employees’ emancipation and to stimulate bold thinking outside of the conventional framework, thus promoting their radical creativity. Therefore, team leaders can encourage empowered employees to think more, with fewer constraints, by fostering a more inclusive and relaxed environment to promote radical creativity. This can be achieved by designing appropriate management practices, such as reducing or even removing punishment for non-subjective errors, encouraging trial-and-error behavior by employees (within a reasonable range), organizing regular meetings to focus on error improvement efforts, and related practical strategies.

### Limitations and future directions

The current study has several limitations. First, the present research explored the influence of empowering leadership on employee radical creativity, responding to researchers’ calls to discuss the effect and mechanism of specific creativity. However, according to the definition, in addition to radical creativity, creativity has another dimension: incremental creativity, which has different definitions and characteristics compared with radical creativity. Therefore, it is also worth exploring the mechanisms that link empowering leadership with incremental creativity. For instance, the factors such as willingness to take risks influencing incremental creativity and radical creativity maybe different, because the risks accompanied with these two forms of creativities would vary. Accordingly, future research could simultaneously investigate the effect of empowering leadership on employee incremental creativity and dual creativity, comparing and contrasting the differences between the mechanisms linking empowering leadership and incremental creativity or radical creativity.

Second, this study suggests that employee traits may also influence the relationship between empowering leadership and employee radical creativity, although this was not specifically explored. For example, previous studies have suggested that employee uncertainty avoidance may affect the impact of empowering leadership on employee creativity and can also influence employees’ risk preferences. Therefore, it can be speculated that employee uncertainty avoidance may also affect the positive effect of employees’ job control on their willingness to take risks. This study suggests that the influence of employees’ traits on the impact of empowering leadership on employee radical creativity should be considered in subsequent research.

Finally, this study mainly explored the positive impact of empowering leadership on employee radical creativity. Although the positive effect of empowering leadership is widely recognized, it should not be ignored that it may also have a “dark” side. Some studies have begun to focus on the possible negative effects of empowering leadership on employees. For example, some scholars have explored the negative effects of empowering leadership, in the form of intimidating passion, on both the routine performance and innovation performance of employees. Therefore, future research should explore any negative impacts of empowering leadership on employees’ breakthrough creativity.

## Data availability statement

The raw data supporting the conclusions of this article will be made available by the authors, without undue reservation.

## Author contributions

WY wrote the manuscript. SL helped with the paper revision and collected the data. All authors contributed to the article and approved the submitted version.

## Funding

This work was supported by the Young Scientists Funds of the National Natural Science Foundation of China (Grant No. 71802073).

## Conflict of interest

The authors declare that the research was conducted in the absence of any commercial or financial relationships that could be construed as a potential conflict of interest.

## Publisher’s note

All claims expressed in this article are solely those of the authors and do not necessarily represent those of their affiliated organizations, or those of the publisher, the editors and the reviewers. Any product that may be evaluated in this article, or claim that may be made by its manufacturer, is not guaranteed or endorsed by the publisher.

## Figures and Tables

**Table:** 

Variables	Measure
Empowering leadership (Ahearne et al., 2005)	1. My leader helps me understand how my objectives and goals relate to that of the company.
	2. My leader helps me understand the importance of my work to the overall effectiveness of the company.
	3. My leader helps me understand how my job fits into the bigger picture.
	4. My leader makes many decisions together with me.
	5. My leader often consults me on strategic decisions.
	6. My leader solicits my opinion on decisions that may affect me.
	7. My leader believes that I can handle demanding tasks.
	8. My leader believes in my ability to improve even when I make mistakes.
	9. My leader expresses confidence in my ability to perform at a high level.
	10. My leader allows me to do my job my way.
	11. My leader makes it more efficient for me to do my job by keeping the rules and regulations simple.
	12. My leader allows me to make important decisions quickly to satisfy customer needs.
Job control (Van Yperen and Hagedoorn, 2003)	1. I can choose the methods to use in carrying out my work.
	2. I can plan my own work.
	3. I can set my own pace.
	4.I can vary how I do my work.
	5. On my job, I have the freedom to take a break whenever I wish to.
	6. I can decide on the order in which I do things.
	7. I can decide when to finish a piece of work.
	8. I have full authority in determining how much time I spend on particular tasks.
	9. I can decide how to go about getting my job done.
	10. My job allows me to organize it by myself.
	11. I have full authority in determining the content of my work.
Willingness to take risks (Schilpzand et al., 2018)	1. I will take an informed risk at work in order to try and get better results.
	2. I will take a risk and try something new that might improve work.
Radical creativity (Madjar et al., 2011)	1. This team member demonstrates a good source of highly creative ideas.
	2. This team member demonstrates originality in his/her work.
	3. This team member suggests radically new ways for doing something.
Error management climate (Cigularov et al., 2010)	1. For us, errors are very useful for improving the work process.
	2. After an error, people think through how to correct it.
	3. Although we make mistakes, we do not let go of the final goal.
	4. An error provides important information for the continuation of the work.
	5. After an error has occurred, it is analyzed thoroughly.
	6. When people are unable to correct an error by themselves, they turn to their co-workers.
	7. If something went wrong, people take the time to think it through.
	8. Our errors point us at what we can improve.
	9. After making a mistake, people try to analyze what caused it.
	10. When an error is made, it is corrected right away.
	11. When people make an error, they can ask others for advice on how to continue.
	12. When working for this contractor, people think a lot about how an error could have been avoided.
	13. When mastering a task, people can learn a lot from their mistakes.
	14. When an error has occurred, we usually know how to rectify it.
	15. If people are unable to continue their work after an error, they can rely on others.
	16. When someone makes an error, he shares it with others so they do not make the same mistake.

## Appendix

Items for all measures.
